# Comprehensive geriatric assessment delivered by advanced nursing practitioners within primary care setting: a mixed-methods pilot feasibility randomised controlled trial

**DOI:** 10.1186/s12877-023-04218-0

**Published:** 2023-08-24

**Authors:** Reza Safari, Jessica Jackson, Louise Boole

**Affiliations:** College of Health, Psychology and Social Care, University of Derby, Kedleston Rd, Derby, DE22 1GB Derbyshire UK

**Keywords:** Frailty, Older Adults, Advanced Nursing Practitioner, Primary Care, Comprehensive Geriatric Assessment

## Abstract

**Background:**

Comprehensive Geriatric Assessment (CGA)is a widely accepted intervention for frailty and can be cost-effective within a primary care setting.

**Objective:**

To explore the feasibility of identifying older adults with frailty and assess the subsequent implementation of a tailored CGA with care and support plan by Advanced Nursing Practitioners (ANPs).

**Methods:**

A mixed-method parallel randomised controlled trial was conducted. Participants were recruited from two General Practice (GP) centres between January and June 2019. Older adults with confirmed frailty, as assessed by practice nurses, were randomised, using a web service, to the intervention or treatment-as-usual (TAU) groups for six months with an interim and a final review. Data were collected on feasibility, health service usage, function, quality of life, loneliness, and participants' experience and perception of the intervention. Non-parametric tests were used to analyse within and between-group differences. *P*-values were adjusted to account for type I error. Thematic analysis of qualitative data was conducted.

**Results:**

One hundred sixty four older adults were invited to participate, of which 44.5% (*n* = 72) were randomised to either the TAU (*n* = 37) or intervention (*n* = 35) groups. All participants in the intervention group were given the baseline, interim and final reviews. Eight participants in each group were lost to post-intervention outcome assessment. The health service use (i.e. hospital admissions, GP/emergency calls and GP/Accident Emergency attendance) was slightly higher in the TAU group; however, none of the outcome data showed statistical significance between-group differences. The TAU group showed a deterioration in the total functional independence and its motor and cognition components post-intervention (*p* < .05), though the role limitation due to physical function and pain outcomes improved (*p* < .05). The qualitative findings indicate that participants appreciated the consistency of care provided by ANPs, experienced positive therapeutic relationship and were connected to wider services.

**Discussion:**

Frailty identification and intervention delivery in the community by ANPs were feasible. The study shows that older adults with frailty living in the community might benefit from intervention delivered by ANPs. It is suggested to examine the cost-effectiveness of the intervention in sufficiently powered future research.

**Trial registrations:**

The protocol is available at clinicaltirals.gov, ID: NCT03394534; 09/01/2018.

**Supplementary Information:**

The online version contains supplementary material available at 10.1186/s12877-023-04218-0.

## Background

The number of people over the age of 85 has nearly doubled in the past three decades and it is estimated that by 2030 one in five people in England will be over the age of 65 [1]. More than 50% of hospital admissions might be associated with frailty [2, 3]. It is a health status related to reduced function across multiple physiological systems which develops due to complex ageing mechanisms. A consensus group has defined frailty as “a medical syndrome with multiple causes and contributors that is characterised by diminished strength, endurance, and reduced physiologic function that increases an individual’s vulnerability for developing increased dependency and/or death” [2]. The best practice guidance, produced by the British Geriatric Society (BGS) [4], recommends assessing older adults for the presence of frailty during all health and social care professionals' visits, using available validated tools, and provision of subsequent necessary care. Despite the BGS’s recommendations offering routine population screening for frailty is challenging because of the low specificity of available tools [5].

Comprehensive Geriatric Assessment (CGA) offers holistic multidisciplinary care for older adults with frailty. CGA takes into account various aspects of a person's health, such as their physical medical condition, mental health, functioning, social circumstances, and environment. This approach is used to create a personalised care plan that involves multiple agencies and disciplines, with specific goals aimed at facilitating discharge planning and minimising unnecessary hospital admissions [6]. A recent review of CGA interventions for persons aged ≥ 65 delivered within primary care settings identified four heterogeneous studies in design and outcome measures showing improvement in adherence to medication modification and acceptance and potential cost-effectiveness of the Interventions, but no improvement in survival or functional outcomes, and presenting mixed results in terms of post-intervention hospital admission rates [7]. In this and other available systematic reviews and meta-analyses, heterogeneity in study design, outcomes, intervention characteristics and or health and care team composition across the included studies have limited the overall conclusion on the effectiveness of CGA delivered in patients' homes [8] or primary care settings [7, 8]. In most cases, the intervention was based on an assessment-recommendation model with a lack of patient adherence to the recommendation and insufficient detailed and consistent descriptions of the interventions and frailty.

Furthermore, accurate and economical screening and identifying suitable candidates for CGA is the first step to avoiding over-or under-prescription. The electronic Frailty Index (eFI) [9] showed predictive validity for the outcome of hospitalisation, nursing home admission and mortality [9]. However, it may not portray the larger picture of frailty as a complex condition [10, 11], and therefore the use of other valid measures of frailty in addition to eFI can potentially improve the identification of individuals at risk of adverse health outcomes [12].

Once the older adults with frailty are identified, a CGA and subsequent care and support plan could be effectively delivered in the community or patient’s home. However, coordination of multidisciplinary team assessment and care and support plan formulation is impractical because of busy General Practice (GP) Centres; therefore, it is not feasible for anyone with frailty to undergo a full multidisciplinary CGA [13]. Nevertheless, these patients could benefit from a holistic review according to the principles of CGA by Advance Nursing Practitioners (ANPs) specialised in caring for older adults [13, 14].

This study aimed to explore the feasibility of identifying frail older adults who benefit most from CGA and subsequent implementation of a tailored CGA with care and support plan (CGA & CSP) for older adults with frailty by ANPs. Additionally, it aimed to measure key health-related outcomes, estimate healthcare resource usage, and evaluate patients’ perception of the intervention. In line with this, the objectives were:

1. To assess the feasibility of the study, which includes reporting the study invitation response rate, the eligibility rate (i.e. the number of frail older adults confirmed by nurse’s assessment and clinical judgement), the recruitment rate (i.e. the number of participants who were eligible and randomised), the retention rate (i.e. the number of participants available for interim and final CGA & CSP review) and the dropout rate (i.e. participants not available for post-intervention outcome assessment).
2. To explore whether the intervention (i.e. CGA & CSP) improves key health-related outcome domains such as function, quality of life, and loneliness (see Deviation from protocol).
3. To estimate the utilisation of health care resource usage and expenses related to CGA & CSP delivery. This includes time ANPs spend on CGA & CSP, as well as hospital and nursing home admission, 999 calls, Accident and Emergency department (A&E) attendance, GP Centre, or GP Centre out-of-hour attendance and calls.
4. To evaluate older adults’ perceptions of the CGA & CSP intervention and delivery.

## Methods

### Design

A mixed-methods parallel randomised controlled trial design with an equal randomisation ratio, stratified based on level of frailty, was conducted. No sample size calculation was performed for this trial. Still, it was estimated that 160 people with frailty would be included based on the resource available and the number of registered patients at two participating GP centres. Those eligible completed the study outcome assessment form and subsequently were randomised to either CGA & CSP or treatment as usual (TAU) care using a web-based randomiser available at randomizer.org by a research assistant. The intervention group received the CGA & CSP for six months, which included an interim (3 months) and a final (6 months) review. Data were collected 2–3 weeks prior to the start of the intervention and again 2–3 weeks after final review. Professionals delivering the intervention to participants were masked to baseline and follow-up measurements. Outcome assessors were also blind to group allocation. A protocol was published a priori at clinicaltirals.gov ID: NCT03394534 on 09/01/2018. To ensure the quality of reporting, the Consolidated Standards of Reporting Trials (CONSORT), an extension to randomised pilot and feasibility trials (2010), was followed.

### Participants’ screening and eligibility

**Fig. 1:**
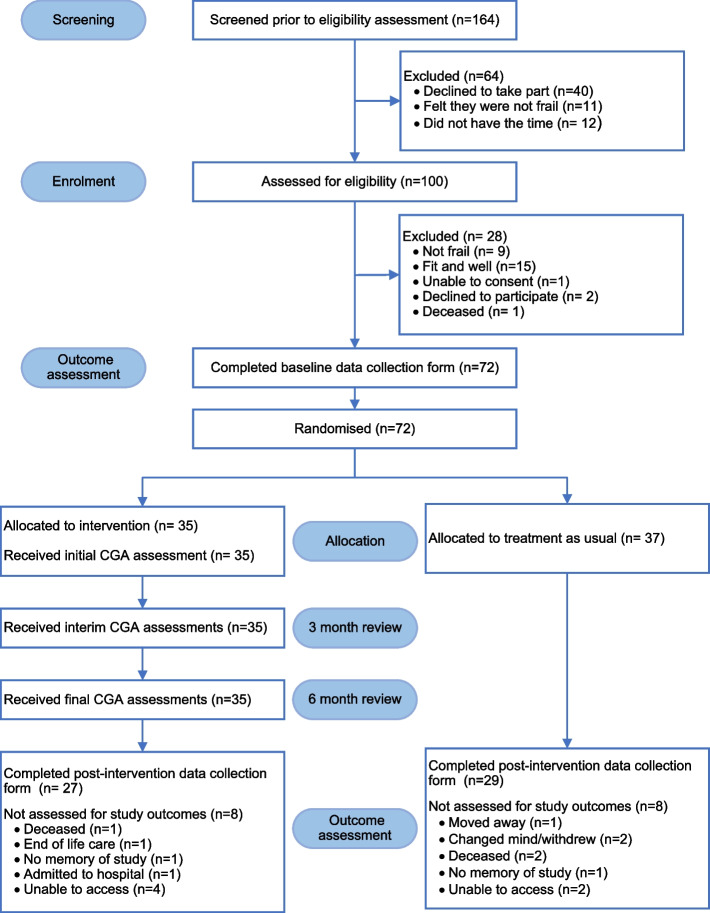
Study flow diagram

To be included in the study, patients had to be assessed by a practice nurse and show mild, moderate, or severe levels of frailty. Between January and June 2019, administrative staff at two GP centres in Belper, UK, screened patients’ health records to identify potential eligible candidates for assessment. They invited all registered patients over 65 with an eFI score of at least 0.12. [9] via telephone. During the telephone conversation, the staff explained the study and outlined the requirements for participation. The participant's information sheet (PIS) and consent form were then posted to participants who expressed interest in participating. Subsequently, a practice nurse visited the patients either at their homes or the GP practice centre, depending on what was most convenient for patients. In the session, the nurse addressed participant queries, obtained informed consent, and evaluated frailty as the primary eligibility criterion. To confirm frailty, the nurse administered timed up-and-go (TUG) test [15], Rockwood [16] and PRISMA-7 (Program of Research on Integration of Services for the Maintenance of Autonomy) [17] scales using a frailty screening form (Additional file 1) [5]. See the “Deviation from the Protocol” section for more information. Although it would have been ideal for every participant to have had a family member or a caregiver to assist them, this was not an inclusion criterion [5]. Participants who were not clinically judged as frail were excluded from the study. The practice nurse supported individuals who were confirmed to have frailty in completing the data collection form for the study. Afterwards, the practice nurse provided the eligible participants' Patient ID to the research assistant at the university for randomisation. For the study flow diagram see Fig. 1.

### Intervention

**Fig. 2:**
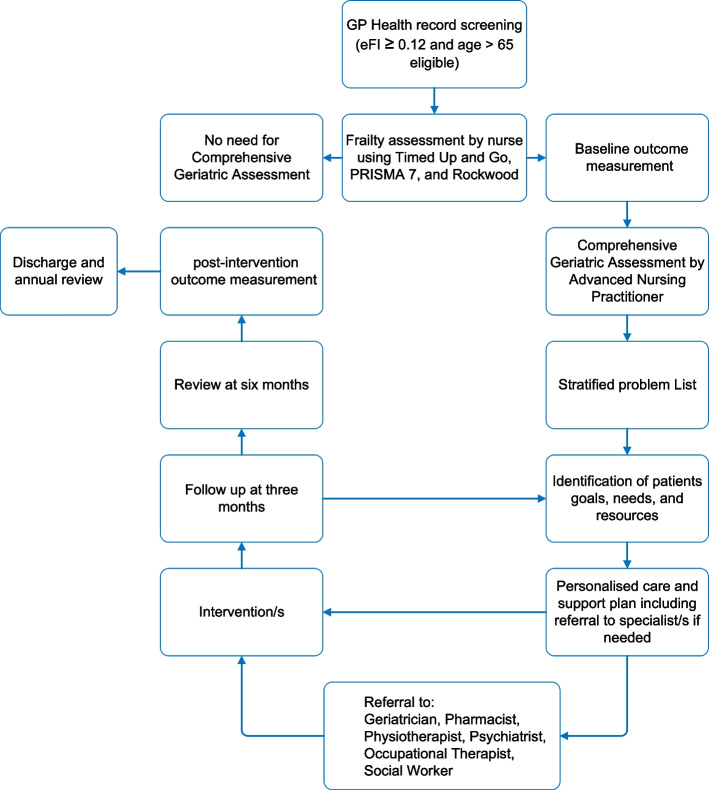
CGA & CSP flow diagram

Two ANPs, specialising in the health and care of older adults, adopted a holistic, biopsychosocial approach to assessing participants at their homes or the GP centres depending on individual participants’ convenience. A CGA proforma with sections on detailed assessment strategy, matters important to participants, referrals, and care and support plan protocol, was developed based on available literature and expert opinion (see Additional file 2). The ANPs produced a personalised goal-oriented care and support plan incorporating a self-care programme in collaboration with the patients and their family members or carers. If needed ANPs referred to other specialists in the discipline and acted as a hub. They shared information with other health and social care staff involved in patient care for improved integrated service, Fig. 2. The same CGA proforma was used in all encounters with participants.

### Data collection

Data were collected at baseline and six months post-intervention from both CGA & CSP and TAU groups. Baseline data collection was conducted by practice nurses at participants' homes. A research nurse collected Post-intervention data over the phone (please see section ‘Deviation from protocol’). A data collection form containing all data items was developed before the start of the study following discussion amongst the team members. Data items and variables included sociodemographic variables, eFI, physical frailty test results, frailty diagnosis, and study outcomes (see secondary outcomes). Individuals responsible for screening and inviting patients were trained to identify and invite eligible participants and systematically record the observations, including reasons for non-participation. The training was also given to nurses at participating GP centres by the lead researcher (RS) about frailty assessment and data collection.

#### Feasibility, retention, and dropouts

- Response rate: percentage of the participants willing to participate who were eligible based on the eFI score.
- Eligibility rate: percentage of consented participants eligible who entered the study based on nurses’ clinical frailty diagnosis.
- Recruitment rate: percentage of invited participants who were eligible and randomised.
- Retention rate: percentage of randomised participants available for interim and follow-up reviews.
- Dropout rate: percentage of randomised participants not completing the post-intervention outcome measure tools.

#### Secondary outcomes

The secondary outcomes included:

- Hospital and nursing home admissions during the past six months recalled by the study participants. This outcome was considered the proposed primary outcome of a future trial.
- Participants reported numbers of 999 calls, A&E attendances, GP Centre or GP Centre out-of-office calls and attendance during the past six months.
- ANPs’ time on CGA & CSP delivery and interim and final reviews, taken from completed CGA proforma.
- Function assessed by Functional Independence Measure (FIM) [18].
- Quality of life assessed by Health-Related Quality of Life- Short form (SF36) [19].
- Loneliness assessed by De Jong-Gierveld loneliness scale [20].

#### Qualitative data

 Qualitative data were collected from a subset of intervention group participants (n=13) by the study research nurse (JJ) via telephone interviews at the end of the study. Feedback from the two ANPs was also taken in meeting sessions with researchers during and after the intervention period. The interview schedule was designed to explore the older adult’s perception and experience of being part of the research project. Participants were asked about their perceived challenges, strength and benefits of the assessment, care and support plan (see Additional file 3).

### Data analysis

Descriptive statistics, including mean (standard deviation; SD or standard error; SE) and raw count (%), are reported for continuous and categorical variables, respectively. The Wilcoxon Signed Ranked test was used to analyse within-group differences. Independent t-test, Mann–Whitney test, and Chi-squared test were used to assess between-group differences where appropriate. Analysis was conducted based on per protocol by excluding participants with missing post-intervention data. In addition, an Intention to treat (ITT) analysis was performed, including all participants. Multiple imputations using the linear regression method with 20 iterations were conducted to account for missing values. The sociodemographic and outcome variables were not significantly different between groups at baseline (Tables 1 and 2); therefore, no adjustments for these variables were made. Benjamini-Hochberg's (1995) procedure was used to diminish the False Discovery Rate as a result of multiple family-wise comparisons and adjust p-values to control for Type 1 error [21]. IBM SPSS, version 28, was used for quantitative data analysis.

**Table 1:** Demographics data for individuals assessed for frailty and for participants randomly allocated to study arms

	**Frail** ^†^**(*n* = 72)**	**Not Frail **^†^ **(*n* = 24)**	***p*-value**	**TAU control group (*n* = 37)**	**CGA group (*n* = 35)**	***p*-value**
**Age (years), Mean (SD)**	82.55 (7.62)	78.33 (6.23)	.010 ^‡^	83.08 (7.41)	82.00 (7.90)	.553 ^‡^
**Gender, n (%)**						
Female	51 (70.8)	10 (41.7)	0.01^§^	26 (70.3)	25 (71.4)	.914 ^§^
Male	21 (29.2)	14 (58.3)		11 (29.7)	10 (28.6)	
**eFI score, Mean (SD)**	0.367 (0.08)	0.312 (0.6)	.002 ^‡^	0.37 (0.09)	0.36 (0.08)	.447^‡^
**eFI Category, n (%)**						
Moderate	36 (50.0)	15 (62.5)	.047 ^§^	19 (51.3)	17 (48.6)	.814 ^§^
Severe	36 (50.0)	5 (37.5)		18 (48.7)	18 (51.4)	
**TUG (Sec), Mean (SD)**	21.86 (1.37)	9.92 (2.04)	.001 ^‡^	22.20 (15.69)	21.50 (11.49)	.838 ^‡^
**PRISMA, Median (IQR)**	4.0 (2)	2.0 (2)	.001^¶^	4.0 (3)	4.0 (3)	.233 ^¶^
**Rockwood, Median (IQR)**	5.0 (2)	2.50 (2)	.001^¶^	4.0 (1)	5.0 (3.00)	.095 ^¶^
**Frailty Diagnosis, n (%)**						
Mild	20 (27.7)	-	-	11 (29.7)	9 (25.7)	.586 ^§^
Moderate	39 (54.2)	-	-	21 (56.8)	18 (51.4)	
Severe	13 (18.1)	-	-	5 (13.5)	8 (22.9)	
**BMI (kg/m2), Mean (SD)**	28.09 (5.59)	28.01 (3.32)	.0.941^‡^	27.92 (5.23)	28.29 (6.08)	.819 ^‡^

*BMI* Body mass index, *CGA* Comprehensive Geriatric Assessment, *eFI* Electronic frailty index, *IQR* Inter quartile range, *SD* Standard deviation, *TAU* Treatment as usual

^†^ Frailty diagnosis was based on physical assessment tests and nurses’ clinical diagnosis. ‘Not frail’ individuals were excluded from the study

^‡^ Independent t-test

^§^ Pearson Chi-squared test

^¶^ Mann–Whitney U test

**Table 2:** Baseline means, standard deviation, group difference, standard error, and *p*-values of study outcome for intervention and TAU groups

	Intervention Mean (SD)	TAU Mean (SD)	Mean Diff. (SE)	*p*-value (Adj.)
**Hospital Admission**^a^	0.51 (1.40)	1.30 (3.13)	0.78 (0.57)	1.00
**Nursing Home Admission**^a^	0.00 (0.00)	0.00 (0.00)	0.00 (0.00)	1.00
**999 Calls**^a^	0.09 (0.28)	0.30 (0.91)	0.21 (0.16)	.817
**A&E Attendance**^a^	0.26 (0.51)	0.43 (0.93)	0.18 (0.18)	1.00
**GP Attendance**^a^	0.43 (1.22)	0.46 (1.37)	0.03 (0.30)	1.00
**GP Calls**^a^	0.29 (0.62)	0.84 (1.80)	0.55 (0.31)	.776
**Total Functional Independence Measure**	112.89 (18.16)	116.86 (16.25)	3.98 (4.07)	.776
- Motor	79.06 (17.09)	83.22 (12.54)	4.16 (3.55)	.776
- Social	53.69 (4.83)	53.16 (7.06)	-0.52 (1.42)	1.00
- Self-Care	38.31 (7.95)	39.76 (5.40)	1.44 (1.61)	.842
- Bladder Bowels	12.43 (2.82)	12.76 (3.14)	0.33 (0.70)	.776
- Locomotion	28.34 (7.62)	30.32 (6.19)	1.98 (1.64)	.842
- Communication	13.74 (0.66)	13.59 (1.99)	-0.15 (0.35)	.842
- Cognition	33.83 (2.53)	33.65 (4.11)	-0.18 (0.80)	1.00
**Total Loneliness**^a^	3.11(2.71)	3.35 (2.76)	0.24 (0.64)	1.00
- Emotion Loneliness^a^	2.06 (1.86)	2.24 (1.83)	0.19 (0.44)	1.00
- Social Loneliness^a^	1.06 (1.19)	1.11 (1.33)	0.05 (0.30)	1.00
**Total SF_36**	47.23 (20.38)	46.42 (17.75)	-0.81 (4.51)	1.00
- Physical function	35.29 (27.63)	35.81 (22.25)	0.53 (5.93)	1.00
- Role Limitation due to Physical health	32.86 (37.75)	28.38 (37.34)	-4.48 (8.86)	1.00
- Role Limitation due to Emotional problems	68.57 (42.74)	72.07 (38.10)	3.50 (9.56)	1.00
- Energy/fatigue	34.43 (21.58)	34.46 (20.06)	0.03 (4.92)	1.00
- Emotional Well-being	72.11 (23.47)	71.68 (20.90)	-0.44 (5.25)	1.00
- Social Functioning	82.86 (24.65)	72.97 (28.34)	-9.88 (6.25)	.776
- Pain	45.71 (27.31)	50.88 (23.86)	5.16 (6.06)	.910
- General Health	43.00 (26.96)	42.41 (21.28)	-0.59 (5.75)	1.00
- Health Change	40.00 (17.36)	31.89 (17.29)	-8.11 (4.09)	.776

*Adj* Adjusted, *A&E* Accident and emergency, *GP* General Practice, *FIM* Functional independence measure, *SE* Standard error, *SD* Standard deviation, *SF-36* Short form 36, *TAU* treatment as usual

^a^Smaller values indicate improvement in the outcome

The qualitative data were analysed using the theoretical flexible Braun and Clarke (2006) approach [22]. The telephone interviews were transcribed verbatim. Two researchers imported the data into NVivo 10 for coding. Both researchers then made parallels with their codes and agreed on the final themes. Pseudonyms have been assigned to the participants and presented in illustrated quotes to demonstrate the relevance to the findings.

### Deviation from protocol

There were a few deviations from the study protocol. Due to physical space limitations in most participants' homes, the gait speed test was replaced with Rockwood. Besides, the post-intervention outcome assessments were conducted by a research nurse over the phone in both study groups due to COVID-19 restrictions. Also, details of care received by the TAU were not collected due to the impact of the COVID-19 pandemic on staff time and availability in the participating GP centres. Due to the same reason, we did not hold focus group sessions with staff/practitioners to collect their views on the intervention; however, an informal discussion with ANPs reflected the findings reported in the qualitative findings section. Moreover, it was planned to assess Self-Reported Pain (e.g., Geriatric Pain Measure Short Form), however; after discussion amongst the team, it was decided not to overwhelm participants with too many outcome measure questions, and therefore it was excluded.

## Results

### Participant details

The baseline demographic and study outcomes data were not significantly different between the two study groups (Tables 1 and 2). The mean (SD) age of participants in the TAU and intervention groups were 83.08 (7.41) and 82.00 (7.90), respectively. There were more female participants overall than males; 70.3% in the TAU group and 71.4% in the intervention group. The mean (SD) eFI score was 0.37 (0.09) and 0.36 (0.08) for the intervention and TAU groups, respectively.

### Feasibility findings

The study recruitment, CGA & CSP and outcome assessments began in January 2019 and concluded in February 2020. One hundred sixty-four older adults with eFI ≥ 0.12 were identified and invited to participate; following reading the PIS, 40 (24.4%) declined to take part without stating a reason, 11 (6.1%) felt not being frail and 12 (7.3%) felt not having time to participate. The remaining *n* = 100 of the eFI-eligible individuals who agreed to participate (Response rate: 61.0%) were assessed for eligibility, of which 24.0 did not meet the frailty criteria based on further nurses’ assessment (Eligibility rate = 76%). Those not meeting the frailty criteria had lower eFI, TUG, PRISMA and Rockwood scores, were more men than women and were younger. Body mass index was not significantly different between frail and non-frail individuals. See Table 1.

Of 164 invited participants, 72 were randomised (Recruitment rate: 44.5%) to either the TAU group (*n* = 37) or the intervention group (*n* = 35), see Fig. 1, for reasons for exclusion. All participants in the intervention group received the initial, interim and final CGA assessments by ANPs (100% retention rate). However, eight participants in each group (Dropout rate: 22.9% intervention and 27.6% TAU) were lost to post-intervention outcome assessment because of reasons stated in Fig. 1. Other than the participants who lost to follow-up at the post-intervention time point, collecting the entire study outcome data was feasible using either in-person or telephone-administered questionnaires. Participants could recall their service usage during the past six months before and after the intervention.

### Secondary outcomes

#### Length of stay at a hospital or nursing home

The average number of nights stayed at the hospital during the past six months slightly increased in both groups at the post-intervention time point (see Tables 3 and 4). Participants in the intervention group spent fewer nights in a hospital than the TAU group (ITT mean difference: 1.95, SE: 1.02); however, the between and within-group differences were not statistically significant (Tables 3, 4 and 5). There were no nursing home admissions in either group.

**Table 3:** Post-intervention mean, mean change-from-baseline, standard deviations, and *p*-values for the study groups – Per protocol analysis

	Intervention (*n* = 27)			TAU (*n* = 29)		
	Mean (SD)	Mean Change from baseline (SD)	*p*-value (Adj.)	Mean (SD)	Mean Change from baseline (SD)	*p*-value (Adj.)
**Hospital Admission**^a^	0.78 (2.10)	0.30 (2.40)	.728	3.14 (6.53)	1.52 (7.06)	.650
**Nursing Home Admission**^a^	0.00 (0.00)	0.00 (0.00)	1.00	0.00 (0.00)	0.00 (0.00)	1.00
**999 Calls**^a^	0.07 (0.27)	0.04 (0.34)	.805	0.45 (0.57)	0.07 (0.92)	.743
**A&E Attendance**^a^	0.07 (0.27)	-0.11 (0.32)	.281	0.41 (0.57)	-0.10 (0.98)	.882
**GP Attendance**^a^	0.67 (1.30)	0.33 (1.39)	.495	0.97 (1.45)	0.38 (2.24)	.291
**GP Calls**^a^	0.26 (0.66)	0.00 (0.78)	1.00	0.69 (0.89)	-0.34 (2.32)	1.00
**Total Functional Independence Measure**	111.41 (18.30)	-3.89 (12.86)	.329	109.59 (21.29)	-6.97 (11.95)	**.043**
- Motor	77.89 (15.85)	-3.19 (9.86)	.329	76.62 (17.30)	-6.55 (11.65)	**.043**
- Social	53.22 (8.28)	-1.11 (8.80)	.876	52.45 (7.80)	-0.83 (2.35)	.242
- Self-Care	37.07 (7.95)	-2.15 (5.81)	.277	36.62 (10.41)	-3.17 (7.56)	.242
- Bladder Bowels	13.04 (2.28)	0.41 (1.67)	.277	12.66 (2.68)	-0.28 (1.69)	.623
- Locomotion	27.78 (7.51)	-1.48 (5.73)	.531	27.45 (6.52)	-3.00 (5.55)	.059
- Communication	13.81 (0.79)	0.00 (0.48)	1.00	13.48 (2.25)	0.00 (0.46)	1.00
- Cognition	33.52 (4.22)	-0.70 (4.28)	1.00	32.97 (4.97)	-0.41 (1.24)	.242
**Total Loneliness**^a^	2.59 (2.98)	0.33 (2.34)	.805	2.90 (3.07)	-0.59 (2.69)	.425
- Emotion Loneliness^a^	1.78 (1.91)	0.37 (1.69)	.495	2.10 (2.06)	-0.34 (1.70)	.427
- Social Loneliness^a^	0.81 (1.33)	-0.04 (1.02)	.951	0.79 (1.35)	-0.24 (1.41)	.561
**Total SF_36**	49.52 (17.68)	-1.70 (17.07)	.951	50.45 (16.47)	5.07 (23.07)	.291
- Physical function	26.11 (25.17)	-13.70 (19.25)	**.018**	33.45 (22.52)	-0.52 (25.19)	1.00
- Role Limitation due to Physical health	59.26 (46.59)	22.22 (56.47)	.277	55.17 (45.50)	27.59 (54.01)	.079
- Role Limitation due to Emotional problems	93.83 (22.72)	19.75 (50.01)	.277	85.06 (31.61)	16.09 (48.49)	.291
- Energy/fatigue	31.85 (18.66)	-4.44 (21.98)	.645	32.07 (19.11)	-1.72 (25.01)	.896
- Emotional Well-being	76.30 (12.35)	-1.48 (17.37)	.805	67.31 (17.34)	-0.41 (20.10)	1.00
- Social Functioning	64.35 (31.91)	-25.00 (30.42)	**.018**	75.00 (25.88)	2.59 (44.62)	.894
- Pain	55.00 (31.09)	9.54 (31.42)	.331	65.60 (26.76)	13.97 (33.70)	.126
- General Health	44.81 (20.73)	-1.48 (18.34)	.631	46.72 (15.25)	2.76 (21.45)	.561
- Health Change	27.78 (22.29)	-12.04 (18.82)	**.041**	29.31 (18.98)	-3.45 (24.75)	.743

*Adj* Adjusted, *A&E* Accident and emergency, *GP* General Practice, *FIM* Functional independence measure, *SD* Standard deviation, *SF-36* Short form 36, *TAU* treatment as usual

^a^Smaller values indicate improvement in the outcome. Bold values denote significant adjusted *p*-values at alpha = 0.05

**Table 4:** Post-intervention mean, mean change-from-baseline, standard deviations, z-scores, and *p*-values for the study groups – Intention to treat analysis

	Intervention (*n* = 35)			TAU (*n* = 37)		
	Mean (SD)	Mean Change from baseline (SD)	*p*-value (Adj.)	Mean (SD)	Mean Change from baseline (SD)	*p*-value (Adj.)
**Hospital Admission**^a^	1.06 (2.09)	0.64 (2.32)	.268	3.00 (5.83)	1.28 (6.29)	.245
**Nursing Home Admission**^a^	0.00 (0.00)	0.00 (0.00)	1.00	0.00 (0.00)	0.00 (0.00)	1.00
**999 Calls**^a^	0.18 (0.37)	0.01 (0.45)	.337	0.42 (0.54)	0.05 (0.87)	.259
**A&E Attendance**^a^	0.11 (0.28)	-0.09 (0.41)	.197	0.37 (0.53)	-0.06 (0.90)	.788
**GP Attendance**^a^	0.77 (1.21)	0.26 (1.40)	.337	0.98 (1.32)	0.21 (2.07)	.082
**GP Calls**^a^	0.33 (0.61)	-0.04 (0.90)	.879	0.66 (0.80)	-0.44 (2.13)	.788
**Total Functional Independence Measure**	111.28 (16.12)	-4.08 (11.35)	.380	109.58 (18.89)	-6.82 (10.61)	**.006**
- Motor	77.26 (14.04)	-3.59 (8.86)	.268	76.81 (15.37)	-6.36 (10.35)	**.008**
- Social	53.36 (7.34)	-1.22 (7.77)	.769	52.53 (6.95)	-0.76 (2.44)	.083
- Self-Care	37.08 (7.06)	-1.69 (5.39)	.197	36.47 (9.32)	-3.13 (6.79)	**.021**
- Bladder Bowels	13.13 (2.02)	0.30 (1.53)	.147	12.51 (2.50)	-0.31 (1.60)	.358
- Locomotion	27.50 (6.75)	-1.86 (5.16)	.380	27.73 (5.89)	-2.50 (5.06)	.082
- Communication	13.75 (0.76)	0.04 (0.53)	.952	13.43 (2.02)	0.00 (0.45)	.208
- Cognition	33.19 (3.97)	-0.59 (3.84)	.552	32.83 (4.46)	-0.61 (1.27)	**.015**
**Total Loneliness**^a^	2.47 (2.73)	0.21 (2.20)	.364	2.78 (2.82)	-0.41 (2.55)	.308
- Emotion Loneliness^a^	1.77 (1.71)	0.24 (1.74)	.526	2.04 (1.94)	0.03 (1.87)	.358
- Social Loneliness^a^	0.83 (1.24)	0.03 (1.00)	.337	0.75 (1.21)	-0.30 (1.31)	.208
**Total SF_36**	49.96 (15.59)	-0.67 (15.18)	.380	50.60 (14.62)	3.92 (20.64)	.208
- Physical function	26.85 (22.11)	-12.57 (17.16)	.197	32.97 (20.08)	-1.94 (22.50)	.612
- Role Limitation due to Physical health	57.80 (41.02)	21.79 (49.55)	.073	56.13 (40.25)	27.04 (47.78)	**.015**
- Role Limitation due to Emotional problems	92.67 (20.05)	20.07 (43.89)	.073	85.56 (28.00)	17.11 (42.87)	.208
- Energy/fatigue	31.86 (16.41)	-3.96 (19.40)	.730	31.86 (16.91)	-1.63 (22.10)	.624
- Emotional Well-being	75.11 (11.07)	-1.52 (15.25)	.814	68.50 (15.60)	-0.47 (17.75)	.358
- Social Functioning	65.61 (28.18)	-22.34 (27.37)	.073	73.82 (23.20)	-0.10 (39.89)	.941
- Pain	56.28 (27.43)	9.65 (27.58)	.197	63.88 (23.86)	13.27 (29.84)	**.033**
- General Health	44.90 (18.20)	-0.54 (16.31)	.852	46.48 (13.72)	1.86 (19.15)	.208
- Health Change	28.39 (19.58)	-10.85 (16.64)	.073	29.30 (16.78)	-4.60 (22.03)	.788

*Adj* Adjusted, *A&E* Accident and emergency, *GP* General Practice, *FIM* Functional independence measure, *SD* Standard deviation, *SF-36* Short form 36, *TAU* treatment as usual

^a^Smaller values indicate improvement in the outcome. Bold values denote significant adjusted p-values at alpha = 0.05

**Table 5:** Post-intervention group mean difference, standard error, and p-values for study outcomes

	Per protocol analysis (*n* = 56)		Intention-to-treat analysis (*n* = 72)	
	Mean diff. (SE)	*p*-value (Adj)	Mean diff. (SE)	*p*-value (Adj)
**Hospital Admission**^a^	2.36 (1.28)	.239	1.95 (1.02)	.295
**Nursing Home Admission**^a^	0.00 (0.00)	1.00	0.00 (0.00)	1.00
**999 Calls**^a^	0.37 (0.12)	.094	0.25 (0.11)	.295
**A&E Attendance**^a^	0.34 (0.12)	.095	0.26 (0.10)	.295
**GP Attendance**^a^	0.30 (0.37)	.582	0.21 (0.30)	.659
**GP Calls**^a^	0.43 (0.21)	.239	0.34 (0.17)	.295
**Total Functional Independence Measure**	-1.82 (5.29)	.842	-1.70 (4.13)	.966
- Motor	-1.27 (4.43)	.842	-0.45 (3.47)	.995
- Social	-0.77 (2.15)	.334	-0.83 (1.69)	.295
- Self-Care	-0.45 (2.47)	.582	-0.61 (1.94)	.794
- Bladder Bowels	-0.38 (0.66)	.842	-0.62 (0.53)	.492
- Locomotion	-0.33 (1.89)	.842	0.24 (1.50)	.995
- Communication	-0.33 (0.44)	.842	-0.32 (0.36)	.794
- Cognition	-0.55 (1.23)	.334	-0.36 (0.99)	.435
**Total Loneliness**^a^	0.30 (0.81)	.842	0.31 (0.65)	.966
- Emotion Loneliness^a^	0.33 (0.53)	.842	0.27 (0.43)	.966
- Social Loneliness^a^	-0.02 (0.36)	.842	-0.08 (0.29)	.966
**Total SF_36**	0.93 (4.57)	.842	0.64 (3.57)	.995
- Physical function	7.34 (6.40)	.520	6.12 (4.99)	.573
- Role Limitation due to Physical health	-4.09 (12.32)	.842	-1.66 (9.58)	.995
- Role Limitation due to Emotional problems	-8.77 (7.32)	.520	-7.11 (5.72)	.659
- Energy/fatigue	0.22 (5.05)	.842	-0.01 (3.93)	.969
- Emotional Well-being	-8.99 (4.00)	.239	-6.60 (3.17)	.295
- Social Functioning	10.65 (7.80)	.582	8.21 (6.10)	.492
- Pain	10.60 (7.78)	.496	7.60 (6.07)	.682
- General Health	1.91 (4.89)	.842	1.58 (3.81)	.995
- Health Change	1.53 (5.55)	.842	0.91 (4.31)	.995

*Adj* Adjusted, *A&E* Accident and emergency, *GP* General Practice, *FIM* Functional independence measure, *SE* Standard error, *SF-36* Short form 36, *TAU* treatment as usual

^a^Smaller values indicate improvement in the outcome

#### Other secondary outcomes

The ANPs spend on average 90.96 (95% CI: 81.66 – 100.28) minutes delivering CGA & CSP at their initial session with participants. The time spent with patients was reduced to Mean (95% CI): 68.71 (57.36 – 80.05) and 58.55 (48.36 – 68.74) minutes during the interim and final review sessions, respectively. The between-group difference in the number of 999 calls, GP/GP out-of-hours attendance, GP/GP out-of-hours calls, and A&E attendance showed a slight improvement in favour of the intervention group (Mean difference; SE: 0.37; 0.12, 0.34; 0.12, 0.30; 0.37, 0.43; 0.21 respectively); however, the between-group differences, as well as the within-group change-from-baseline, were not statistically significant for these outcomes, (see Tables 3, 4 and 5).

Physical function, social function, and health change subscales of the SF-36 significantly deteriorated in the intervention group according to per-protocol analysis (p-values: 0.08, 0.018, 0.041 respectively; Table 3). However, the intention to treat analysis did not show a significant change from the baseline for any of the study outcomes in the intervention group. In the TAU group, per protocol analysis revealed a significant decrease in total FIM (*p* = 0.043) and its motor subscale (*p* = 0.043) at the post-intervention time point, Table 3. In the ITT analysis, in addition to these two scores (i.e. total FIM, *p* = 0.006, and motor subscale, *p* = 0.008), the cognition subscale of FIM also showed a significant reduction (*p* = 0.015); however, the role limitation due to physical function (*p* = 0.015) and pain (*p* = 0.033) subscales of the SF-36 showed significant improvement at post-intervention in the TAU group (Tables 3 and 4). No significant between-group differences were discovered in per protocol or ITT analyses, Table 5.

### Contents of the CGA & CSP intervention

The summary of problems identified included vision problems (*n* = 9), Polypharmacy (i.e. ≥ five medications; *n* = 30), fall history (*n* = 13), difficulty with balance (*n* = 17), and risk of fracture due to osteoporosis (*n* = 24). Besides, major problems identified were diabetes, fractures, diverticulitis, cancer, osteoarthritis/arthritis, hypertension and high blood pressure. Other problems included allergies/sensitivities such as hay fever, rashes and intestine/indigestion problems.

As part of the care and support planning, participants were asked to answer questions such as ‘What is important to me’ and ‘What makes life meaningful for me’. Most participants stated that family and friends are the most important things that make life meaningful. The importance of independent living and leisure activities were also emphasised, and therefore, being more active, in good health and being at home was what the participants wished for in the future.

Referrals were made for 18 (53%) participants at the initial CGA session to GP (*n* = 5), ANP (*n* = 8), Occupational Therapy (*n* = 1), community Matron (*n* = 2), Falls prevention (*n* = 2) and care co-ordinator (*n* = 1). Also, referrals were made for 9 (24%) and 13 (35%) participants after the interim and final follow-up CGA sessions, respectively. No harm was reported. For the complete list of problems identified and care and support plans delivered, see Additional file 4.

### Qualitative findings

A total of 13 patients took part in the qualitative interviews (six and seven from each practice). These interviews lasted between 20-45 min.

#### Theme 1: Appreciation for consistent care

There was a strong consensus that receiving the study intervention highlighted that their usual care meant they were often not able to get an appointment when needed, not able to see the same health professional or doctor each time they visited, and felt rushed in an appointment during usual care were concerns. For example, Barbara stated, *‘I got the opportunity to ask, you know, if there was anything, she asked if I missed out or anything I was concerned about’.* Here she felt that the APN were able to spend time asking about her health and her needs and gave her the opportunity to respond. This was greatly appreciated. For another participant, this consistency in care was particularly highlighted as beneficial for older adults. *‘I think to keep in contact with anyone who’s elderly and perhaps that you could see the same person, you know, particularly when you are old’,* Ivy*.*

#### Theme 2: The therapeutic relationship

The patients appreciated that they could build up a rapport with the ANPs during the CGA & CSP programme. Participants felt prioritised as the ANPs provided space for them to ask any questions about their health and therefore ANPs were more aware of their needs. For example, one participant described enjoying his visits with one of the APNs *‘Oh, she was good as gold. We had a laugh half the time, and yeah, no she was very good; I actually miss her coming’* Jon. There was also a strong consensus that the patients felt the APNs cared and this improved the quality of the services. For example, *‘You know, I found that important because she obviously cared. You know she wasn’t just coming in, go through a set of stuff and going again you know what I mean she clearly cared and did everything she could to help’,* Gladys*.* For this participant, it was not just an assessment exercise. However, there was an agreement between ANPs and participants that a wide range of assessments was needed so that the ANPs could gain a holistic picture of what was going on for the patients. One participant added ‘*It’s good to have confidence in the person that you’re dealing with’* Michael*.* For Michael, the combination of the assessments, rapport and competence of the APNs was greatly appreciated.

#### Theme 3: Connecting wider services

The ANPs were able to follow up enquiries for the patients with the surgeries, make further referrals and act as a liaison for follow-up care. For example, ‘*Yeah, she got the occupational therapists in to help me, and you know she referred me to different to other things as well; she was very very good to be honest’,* Jack*.* For this participant, it was the link with wider health services that the APN was able to make, which was beneficial. Another participant stated *‘she was the one who told me about the falling clinic and sorted that out for me’,* Shirly*.* For this participant, she was informed about services they were unaware of, and the APN was able to act proactively in prioritising their needs. It was this proactive care which again was expressed here for Pete *‘things she did that would help you know sort of thing just whatever she thought she could do to help me, she did’.* Again*,* this demonstrates the value of these types of assessments that identify needs and risks as a preventative measure for ill health rather than reactionary.

When asked how participants would rate the quality of the care they received on a scale of 0–10, all patients gave an eight or above.

## Discussion

The study aimed to assess the feasibility of identifying older frail adults living in the community and pilot subsequent CGA & CSP delivery by ANPs. Of 164 individuals identified at the eFI screening stage, 44.5% (*n* = 72) participated in the study. The CGA delivery was acceptable to both participants and the ANPs. The size and reasons for dropouts were similar between groups and were unlikely linked to group assignments.

The target recruitment number for this study was 160 participants; however, only 72 patients were recruited. Most patients were recruited from one of the GPs (n = 63). The low-recruiting GP centre had a period of staff shortage and did not progress according to the plan. However, the higher recruiting GP centre had one consistent administrative staff who indicated their use of communication techniques to engage and build a rapport with patients during the recruitment telephone call. This could have been key to this centre's success because frailty can carry a negative stigma associated with the end of life [23]. For any future study, sufficient time, resources, and training for administrative staff should be provided to improve recruitment.

Fifteen per cent of eFI-eligible individuals were not clinically eligible to enter the study. Even some of those who were diagnosed as frail and entered the study expressed in their interview that they believed they were not ‘frail’ or ‘old’ enough to take part. This contributes to the debate on the conceptual ambiguity of frailty and that when presented as a measure of true discourse, it can be problematic [24]. The eFI score may not recognise the subjective lived experience of the patients' physical, psychological, and social vulnerabilities. Many definitions and operationalisations of frailty exclude psychological and social factors linked to frailty [11]. There are several frailty assessment tools available; however, the relationship between the current methods of frailty assessment and the subsequent management of frail older adults in the community is not well established [25]. A review of frailty instruments based on health records (e.g. eFI) showed that non-clinical determinants of health such as social, behavioural and environmental factors are often missing [26]; even though the less affluent older adults with a lower level of social support and education tend to follow a steeper frailty trajectory [27]. In our study, nurse practitioners' clinical judgement and frailty assessment after eFI screening and the subsequent CGA & CSP revealed these additional aspects of health and well-being. However, there was also a consensus among the interviewed participants and the ANPs that the programme might not be needed for a wider population but for those with more complex needs. This highlights the importance of taking a more holistic approach to frailty diagnosis, care, and early intervention by primary care services to identify the right people for CGA intervention.

Although available clinical guidelines consistently recommend frailty identification, assessment and management strategies, implementing these strategies is challenging and dependent upon individual patient characteristics, circumstances and contextual factors and the level of resources and support available [28]. It has been suggested that interventions should target more markers of frailty, including cognitive, psychological and social well-being [29]. Although most CGA programmes applied the principles of integrated care, social care professionals have been less frequently involved in the assessment and development of care plans [30]. Therefore, one approach for cost-effective delivery of CGA in the community could include a close working relationship between the patient, ANP, and a social worker who can utilise community resources and collaborate with primary care practitioners and multidisciplinary teams [14].

Although the outcomes did not reach statistical significance to show group differences, the health service use (i.e., hospital admissions, GP/999 calls and GP/A&E attendance) was higher in the TAU group compared to the intervention group at the end of the study. The TAU group showed a deterioration in FIM total and motor and cognition subscales post-intervention, though the role limitation due to physical function and pain outcomes improved. In the intervention group, the ITT analysis revealed that the outcome measures did not differ significantly from the baseline. A sample size calculation using SPSS, acquired from the mean (SD) hospital admission of 1.06 (2.09) and 3.00 (5.83) for the intervention and TAU groups, respectively, and power (1 – β) of 0.8, revealed a total of 164 (α = 0.05) or 244 (α = 0.01) participants are needed.

The qualitative findings indicate that ANPs provided consistent quality care by spending time to become more aware of the health and care needs and preferences of participants, building a strong relationship with them, signposting to wider services, and acting as a liaison for follow-up care. This would give a more accurate assessment of their needs and further follow-ups. A review of both qualitative and quantitative studies identified the essential components of implementing person-centred care for older adults, including knowing the older patient as a person, building a relationship of trust, working in an interprofessional team with and for the patient, co-creation of tailored health and care plan, and empowering the person [31]. There is a need for a single point of contact for people with frailty. This could be an ANP who can better understand patients' needs and preferences to act as a care coordinator for patient-centred care. However, considering the limited resources, it may not always be possible because ANPs in this study spend on average about 90 min with each participant in their first session and about 60 min in the subsequent follow-up sessions. Alternatively, an effective and practical method of sharing an individual’s care preference is required to ensure the patient’s choice and preference are recorded and shared for each ‘domain’ of care, including physical, medical, functional, mobility, psychological, socio-economical, and environmental needs.

The post-intervention outcome assessments were conducted by a research nurse over the phone in both study groups. However, the bias attributable to the mode of data collection is considered minimal [32]. Another limitation of the study was, as a condition of ethical approval, the exclusion of people with cognition problems and those who were unable to give consent. Furthermore, the study was delivered in Belper, an area that is ranked at the higher decile of the Multiple Deprivation Index. Therefore, the study participants may not be representative of frail older adults living in the community.

Sufficiently powered studies with a health economic evaluation are required before the approach proposed in this study is adopted on a larger scale. The intervention was delivered to older adults with mild, moderate, or severe levels of frailty over a period of 6 months, and this may be a reason why the secondary outcomes did not demonstrate significant changes. For a further study, it is recommended to target older adults with moderate or severe levels of frailty who may benefit more from the CGA & CSP. Additionally, extending the intervention delivery time to over 12 months with a 6-month interim review could be beneficial.

## Conclusion

The study findings indicate that frailty identification and assessment in the community by nursing practitioners was feasible. This helps to target intervention to older adults living in the community who benefit most from CGA and subsequent care and support plans. CGA & CSP delivery by ANPs was acceptable to participants, and the ANPs were able to get to know the patients and therefore were more aware of their needs. The CGA & CSP might reduce health and care usage and improve functional outcomes in community-dwelling older adults with frailty. However, larger-scale studies with embedded health economic evaluation are required to assess the approach employed in this study.

### Supplementary Information

**Additional file 1.** 

**Additional file 2.** 

**Additional file 3.** 

**Additional file 4.** 

## Data Availability

The datasets used and/or analysed during the current study are available from the corresponding author on reasonable request.

## Abbreviation

ANPs: Advance Nursing Practitioners
A&E: Accident and Emergency
BGS: British Geriatric Society
CGA: Comprehensive Geriatric Assessment
CGA & CSP: Comprehensive Geriatric Assessment with care and support plan
CONSORT: Consolidated Standards of Reporting Trials
eFI: Electronic Frailty Index
FIM: Functional Independence Measure
GP: General Practice
PRISMA: Program of Research on Integration of Services for the Maintenance of Autonomy
SD: Standard deviation
SE: Standard error
SF-36: Short Form 36
TAU: Treatment as usual
TUG: Timed up and go
ITT: Intention to Treat

## References

[1] ONS: Ageing in the UK Datasets 1992 – 2037. In. United Kingdom; 2015.

[2] Morley JE, Vellas B, van Kan GA, Anker SD, Bauer JM, Bernabei R, Cesari M, Chumlea WC, Doehner W, Evans J (2013). Frailty consensus: a call to action. J Am Med Dir Assoc.

[3] Clegg A, Young J, Iliffe S, Rikkert MO, Rockwood K (2013). Frailty in elderly people. Lancet.

[4] British Geriatrics Society: Fit for Frailty - consensus best practice guidance for the care of older people living in community and outpatient settings In.: British Geriatrics Society 2014.

[5] Clegg A, Rogers L, Young J (2015). Diagnostic test accuracy of simple instruments for identifying frailty in community-dwelling older people: a systematic review. Age Ageing.

[6] Banerjee J, Conroy S, O’Leary V, Rawstorne S, Wenman J, Walker A, Docherty M, Donovan S, Mukherjee S, Strurgess I (2012). Silver Book: Quality care for older people with urgent and emergency care needs.

[7] Garrard JW, Cox NJ, Dodds RM, Roberts HC, Sayer AA (2020). Comprehensive geriatric assessment in primary care: a systematic review. Aging Clin Exp Res.

[8] Pritchard C, Ness A, Symonds N, Siarkowski M, Broadfoot M, McBrien KA, Lang E, Holroyd-Leduc J, Ronksley PE: Effectiveness of hospital avoidance interventions among elderly patients: A systematic review. CJEM. 2020, 22(4):504–513. 10.1017/cem.2020.4 32216860

[9] Clegg A, Bates C, Young J, Ryan R, Nichols L, Ann Teale E, Mohammed MA, Parry J, Marshall T (2016). Development and validation of an electronic frailty index using routine primary care electronic health record data. Age Ageing.

[10] Nghiem S, Sajeewani D, Henderson K, Afoakwah C, Byrnes J, Moyle W, Scuffham P: Development of frailty measurement tools using administrative health data: A systematic review. Arch Gerontol Geriatrics 2020, 89. 10.1016/j.archger.2020.104102 32464423

[11] Van Damme JK, Lemmon K, Oremus M, Neiterman E, Stolee P (2021). Understanding Frailty Screening: a Domain Mapping Exercise. Can Geriatr J.

[12] Rockwood K (2016). Screening for grades of frailty using electronic health records: where do we go from here?. Age Ageing.

[13] British Geriatrics Society: Fit for Frailty - Part 2: Developing, commissioning and managing services for people living with frailty in community settings In.: British Geriatrics Society 2015.

[14] Pilotto A, Cella A, Pilotto A, Daragjati J, Veronese N, Musacchio C, Mello AM, Logroscino G, Padovani A, Prete C (2017). Three decades of comprehensive geriatric assessment: evidence coming from different healthcare settings and specific clinical conditions. J Am Med Dir Assoc.

[15] Savva GM, Donoghue OA, Horgan F, O’Regan C, Cronin H, Kenny RA (2012). Using timed up-and-go to identify frail members of the older population. J Gerontol.

[16] Rockwood K, Song X, MacKnight C, Bergman H, Hogan DB, McDowell I, Mitnitski A (2005). A global clinical measure of fitness and frailty in elderly people. CMAJ.

[17] Hébert R, Dubois MF, Raîche M, Dubuc N: The effectiveness of the PRISMA integrated service delivery network: preliminary report on methods and baseline data. In: Integration of services for disabled people: research leading to action. Edited by Hébert R, Tourigny A, Raîche M, vol. II. Québec, Canada: EDISM; 2008: 216. PMC2254488 18317561

[18] Ottenbacher KJ, Hsu Y, Granger CV, Fiedler RC (1996). The reliability of the functional independence measure: a quantitative review. Arch Phys Med Rehabil.

[19] Walters SJ, Munro JF, Brazier JE (2001). Using the SF-36 with older adults: a cross-sectional community-based survey. Age Ageing.

[20] Gierveld JDJ, Tilburg TV (2006). A 6-item scale for overall, emotional, and social loneliness: Confirmatory tests on survey data. Res Aging.

[21] Benjamini Y, Hochberg Y (1995). Controlling the false discovery rate: a practical and powerful approach to multiple testing. J Roy Stat Soc: Ser B (Methodol).

[22] Braun V, Clarke V (2006). Using thematic analysis in psychology. Qual Res Psychol.

[23] Gilleard C, Higgs P. Frailty, disability and old age: a re-appraisal. Health. 2011; 15(5):475–490. 10.1177/1363459310383595 21169203

[24] Tomkow L (2020). The emergence and utilisation of frailty in the United Kingdom: a contemporary biopolitical practice. Ageing Soc.

[25] Tolley APL, Ramsey KA, Rojer AGM, Reijnierse EM, Maier AB (2021). Objectively measured physical activity is associated with frailty in community-dwelling older adults: A systematic review. J Clin Epidemiol.

[26] Bery AK, Anzaldi LJ, Boyd CM, Leff B, Kharrazi H (2020). Potential value of electronic health records in capturing data on geriatric frailty for population health. Arch Gerontol Geriatr.

[27] Welstead M, Jenkins ND, Russ T, Luciano M, Muniz-Terrera G. A systematic review of frailty trajectories: their shape and influencing factors. the gerontologist. 2020. 10.1093/geront/gnaa061 PMC8599181 32485739

[28] Mehta P, Lemon G, Hight L, Allan A, Li C, Pandher SK, Brennan J (2021). A systematic review of clinical practice guidelines for identification and management of frailty. J Nutr Health Aging.

[29] Puts MTE, Toubasi S, Andrew MK, Ashe MC, Ploeg J, Atkinson E, Ayala AP, Roy A, Rodríguez Monforte M, Bergman H (2017). Interventions to prevent or reduce the level of frailty in community-dwelling older adults: a scoping review of the literature and international policies. Age Ageing.

[30] Stoop A, Lette M, Gils PF, Nijpels G, Baan CA, Bruin SR. Comprehensive geriatric assessments in integrated care programs for older people living at home: a scoping review. Health Soc Care Community. 2019. 10.1111/hsc.12793 PMC6852049 31225946

[31] Ebrahimi Z, Patel H, Wijk H, Ekman I, Olaya-Contreras P (2021). A systematic review on implementation of person-centered care interventions for older people in out-of-hospital settings. Geriatr Nurs.

[32] Rutherford C, Costa D, Mercieca-Bebber R, Rice H, Gabb L, King M: Mode of administration does not cause bias in patient-reported outcome results: a meta-analysis. Qual Life Res. 2015, 25. 10.1007/s11136-015-1110-8 26334842

