# Supplementary material for: Comprehensive geriatric assessment delivered by advanced nursing practitioners within primary care setting: a mixed-methods pilot feasibility randomised controlled trial
Source: BMC Geriatr. 2023 Aug 24;23:513. doi: 10.1186/s12877-023-04218-0 (PMC10463370; doi:10.1186/s12877-023-04218-0)
Supplement: Supplementary file 1 — Additional file 1. [file 12877_2023_4218_MOESM1_ESM.docx]

# Comprehensive Geriatric Assessment Delivered by Advanced Nursing Practitioners within Primary Care Setting: A Mixed-methods Pilot Feasibility Randomised Controlled Trial

# Additional File 1: Clinical Frailty Screening

To be completed by the Practice Nurse

# Patient ID

## Patient ID: …………………………………………………

## Date of Birth: …………………………………………………

# Frailty Screening

## Electronic Frailty Index (eFI) score

## Timed get up and go Score

## PRISMA 7:

### Are you more than 85 years?

### Male?

### In general do you have any health problems that require you to limit your activities?

### Do you need someone to help you on a regular basis?

### In general do you have any health problems that require you to stay at home?

### In case of need can you count on someone close to you?

### Do you regularly use a stick, walker or wheelchair to get about?

### Prisma 7 total Score:

- 1. Rockwood Score

## **Frailty diagnosis***:

### Mild

### Moderate

### Severe

### Not frail (Fit and Well)

*** Exclude from the study if not frail.** If frail fill below.

Weight (kg): Height (cm):

Waist (cm)
