# Supplementary material for: Comprehensive geriatric assessment delivered by advanced nursing practitioners within primary care setting: a mixed-methods pilot feasibility randomised controlled trial
Source: BMC Geriatr. 2023 Aug 24;23:513. doi: 10.1186/s12877-023-04218-0 (PMC10463370; doi:10.1186/s12877-023-04218-0)
Supplement: Supplementary file 2 — Additional file 2. [file 12877_2023_4218_MOESM2_ESM.docx]

# Comprehensive Geriatric Assessment Delivered by Advanced Nursing Practitioners within Primary Care Setting: A Mixed-methods Pilot Feasibility Randomised Controlled Trial

# Additional File 2: CGA&CSP form

[1) Clinical Frailty Full Assessment 2](#_Toc138165210)

[2) Summary and Problem List 2](#_Toc138165212)

[3) Physical Health Prompt: Offer a cardiovascular and respiratory examination 3](#_Toc138165213)

[4) Medications 7](#_Toc138165214)

[5) Bone Health and Falls Assessment 8](#_Toc138165215)

[6) Function, Social and Environmental Assessment 9](#_Toc138165216)

[7) Mobility and Balance 12](#_Toc138165217)

[8) Psychological Health 12](#_Toc138165218)

[9) Care and Support Plan 14](#_Toc138165219)

# Clinical Frailty Full Assessment

To be completed by Advanced Nursing Practitioner

**Patient ID**

Patient ID: …………………………………………………

Date of Birth: …………………………………………………

When completed, ACP to record the time spent performing CGA: …………………….(min)

Initial Review

Interim Review

Final Review

# Summary and Problem List

- 1. Allergies/Sensitivities:
  2. Active Major Problems
  3. Under care of.. (e.g.: general medical practice, District nurse, community matron, mental health team, etc)

1. Physical Health
   Prompt: Offer a cardiovascular and respiratory examination

| - 1. **Cardiovascular**   Manually check pulse. Report any new irregularities to GP or refer to ECG. Patient should lay down for 10 mins before check lying BP. A drop of > 20 mmHG systolic bp or >10mmHg diastolic or light-headedness or dizziness is considered abnormal. If patient has postural hypotension then subsequent readings should be with the patient standing.  **Note:** If using an automated blood pressure monitoring device, ensure that the device is validated (3) and an appropriate cuff size for the person’s arm is used.  Average blood pressure target (NICE 2011) Below 140/90 mmHg in people aged under 80 years with treated hypertension Below 150/90 mmHg in people aged 80 years and over, with treated hypertension  Sitting systolic blood pressure mmHg  Lying systolic blood pressure mmHg  Standing systolic blood pressure mmHg  Pulse rate bpm  Pulse Rhythm |
| --- |

- 1. **Respiratory**

MRC Breathlessness Scale: This scale measures perceived respiratory disability. The scale is simple to administer as it allow the patient to indicate the extent to which their breathlessness affects their mobility. The 1-5 stage scale is used alongside the questionnaire to establish clinical grades of breathlessness. Used with permission of the Medical Research Council

Grade 1: Is the patients breath as good as that of other men of his age and build at work, on walking, and on climbing hills or stairs?

Grade 2: Is the patient able to walk with normal men of own age and build on the level but unable to keep up on hills or stairs?

Grade 3: Is the patient unable to keep up with normal men on the level, but able to walk about a mile or more at his own speed?

Grade 4: Is the patient unable to walk more than about 100 yards on the level without a rest?

Grade 5: Is the patient breathless on talking or undressing, or unable to leave his house because of breathlessness?

Record MRC Breathlessness Scale score (Grade)

**Central Nervous System Observation**

| - 1. **Nutrition** MUST Screening Tool | | | | |
| --- | --- | --- | --- | --- |
| (YHCS Malnutrition Universal Screening Tool MUST) score | | | |  |
| Step 4:  Overall Risk of Malnutrition   \| **Score** \| **Risk** \| \| --- \| --- \| \| 0 \| Low risk \| \| 1 \| Medium Risk \| \| 2 or more \| High Risk \| | Step 1 – BMI range | | |  |
|  | Step 2 - Unplanned Weight Loss Score | | |  |
|  | Step 3 –Acute Disease Effect Score | | |  |
|  | Step 3 – Acute Disease Effect Score | | |  |
| **Weight management advice needed** | |  | **Dietary advice needed** |  |

- 1. **Bladder & Bowel**
     Prompt: Check Skin Integrity (including skin condition of legs & feet)

Continence assessment

- 1. **Oral Health**

| Swallowing Difficulty |  | | |  | Dental |  |
| --- | --- | --- | --- | --- | --- | --- |
| Oral Health advice needed | |  |  |  |  |  |

- 1. **Feet**Prompt: Refer to Podiatrist. Advise re sensible foot wear

| O/E – general skin exam | |
| --- | --- |
| O/E – foot abnormal | O/E – inappropriate footwear |

- 1. **Sleep**Sleep Pattern/Problems

**k) Alcohol and Smoking**Smoking Status and Intervention Offered

| Patient Smoking Status:   \| Smoker \|  \| \| --- \| --- \| \| Cigarette per day consumption \|  \| \| Ex-Smoker \|  \| \| Never Smoked tobacco \|  \| \| Refusal to give smoking status \|  \| | \| Smoking Intervention Declined: \| \| \| --- \| --- \| \| Not interested in stopping \|  \| \| Family member not interested in stopping \|  \| \| Smoking cessation provided previously \|  \| \| Smoking Cessation Intervention: \| \| \| Brief intervention for stopping offered \|  \| \| Referral to stop-smoking services \|  \| | Alcohol Status:   \| Teetotaller \|  \| \| --- \| --- \| \| Light drinker 1-2 u/day \|  \| \| Moderate drinker 3-6 u/day \|  \| \| Heavy drinker 7-9 u/day \|  \| \| Very heavy drinker 9 + u/day \|  \| \| Self-Referral to alcohol team \|  \| |
| --- | --- | --- | --- | --- | --- | --- | --- | --- | --- | --- | --- | --- | --- | --- | --- | --- | --- | --- | --- | --- | --- | --- | --- | --- | --- | --- | --- | --- | --- | --- | --- | --- | --- | --- | --- | --- | --- | --- |

**l) Vision** (Poor vision, Sight Not tested for 12 months, Varifocals/Bifocals Worn)
Eye and vision observation

|  |
| --- |

If any observations recorded:
Refer to optician. Remember the household will need a domiciliary/visit

|  |
| --- |

Visit treatment – Advise see optician

**m) Hearing**

| Wears a hearing aid? | Yes |  | No |  |  |  |
| --- | --- | --- | --- | --- | --- | --- |
| If yes – does the patient have the hearing aid with them? | Yes |  | No |  |  |  |
| If yes | Right? |  | Left? |  | Both? |  |

Hearing observation. Any issues identified?

| Referral to audiology clinic | Yes |  | No |  |  |  |
| --- | --- | --- | --- | --- | --- | --- |

**n) Accessible Information**

**Please record any access issues or requirements that the patient has, detailing the need and the patient suggestion for improved access.** If the patient has no additional communication requirements please tick **Able to communicate needs and wishes

Communication support**

| Does used hearing aid |  |  | Able to communicate needs and wishes |  |
| --- | --- | --- | --- | --- |
| Uses sign language |  |  | **Specific contact method** |  |
| Using British Sign Language |  |  | Requires contact be telephone |  |
| Using lip-reading |  |  | Requires contact by short message service text |  |
| Uses manual note taker |  |  | Requires contact by letter |  |
| Uses legal advocate |  |  | Requires contact by email |  |
| Uses citizen advocate |  |  | Requires contact by text relay |  |
| Using Makaton sign language |  |  | Requires audible alert |  |
| Uses speech to text reporter |  |  | Requires visual alert |  |
| Uses cued speech transliterator |  |  | Requires tactile alert |  |
| Uses lipspeaker |  |  | |  |
| Uses communication device |  |  |  |  |
| Uses deafblind intervener |  |  |  |  |
| Uses textphone |  |  |  |  |
| Uses electronic note taker |  |  |  |  |
| Record preferred contact method if stated and offer Online services for patients and/or email/SMS – receiving communication in electronic format allows patients to use text on screen readers | | | | |
| Record Language |  |  | | |
| Record Contact details |  |  | | |
| Register for Online Services |  |  | | |

**Specific info format**

| Requires information in Easyread |  |  | Communication professional |  |
| --- | --- | --- | --- | --- |
| Requires information verbally |  |  | Interpreter needed |  |
| Reqs written info in at least 20 point sands serif font |  |  | Requires deafblind communicator guide |  |
| Reqs written info in at least 24 point sands serif font |  |  | Needs an advocate |  |
| Reqs written info in at least 28 point sands serif font |  |  | Requires manual note taker |  |
| Requires information in uncontracted (Grade 1 Braile) |  |  | Requires lipspeaker |  |
| Requires information in uncontracted (Grade 2 Braile) |  |  | Interpreter needed – Makaton Sign Language |  |
| Requires information on compact disc |  |  | Sign Supported English interpreter needed |  |
| Requires information on audio cassette tape |  |  | Requires deafblind manual alphabet interpreter |  |
| Requires information in electronic audio format |  |  | Requires deafblind block alphabet interpreter |  |
| Requires information in Moon Alphabet |  |  | Requires deafblind haptic communication interpreter |  |
| Requires information in Makaton |  |  | Visual frame sign language interpreter needed |  |
| Requires information on USB mass storage format |  |  | Hands-on signing interpreter needed |  |
| Requires information in electronic downloadable format |  |  | Requires speech to text reporter |  |
| Requires information by email |  |  |  | |
| Requires information by telephone |  |  |  | |

# Medications

- 1. Last medication review date and who by
  2. Medications History *(a. Primary care prescription; b. Pharmacy dispensation history; c. Prescribed medications from other providers (eg private healthcare, from abroad); d. Other medications taken (eg leftover tablets, medicines prescribed for others); e. Herbal supplements, vitamins etc; f. Illicit drugs)*
  3. Administration of medication and prescriptions
- Self-administration of medication
- Does not mange medication
- Does manage medication
- Uses dispensed monitored dosage system
- Uses monitored dosage system
- Prescription collected by patient
- Prescription collected by pharmacist
- Prescription collected by family member
  1. Medication review (*consider high risk medications e.g. sedatives, anti-psychotics, NSAIDs, insulin, anti-coagulants, antihypertensives in fallers*):
     1. General:
- Are you good at remembering your pills?
- Can you swallow them OK?
- What are you most concerned about with your tablets?
  - 1. Specific (*For each medication: “Do you take this?”; “How often?”; “What for?”; “Do you think it works?”; “Does it have any side effects?”)*
  1. Medication considerations in acutely unwell patients (*Acute Kidney Injury, Chronic Kidney Disease, hot weather advice on hydration* )
  2. Pharmacological interactions or common side effects (*The STOPP list or equivalent is useful here, as well as the BNF or e-prescribing decision support to check for interactions. This will guide further questioning*)
  3. Appropriateness of medication and patient understanding of medication given the medical history and current disease status *(MAI and STOP can be useful here)*
  4. List and prioritise medication-related issues and discuss changes with the patient
- *Any high-risk prescribing should be changed urgently*
- *Patient priorities are very important to both guide the process and build trust*
- *Changes should generally be introduced progressively over time unless there is a significant, urgent problem*
- *New medications should also usually be introduced one-by-one (to avoid confusion if prescribing or de-prescribing causes new symptoms)*
- *Reduce old medications gradually if necessary to avoid rebound effects (physiological or psychological dependence) and introduce new medications gradually too – start low and go slow.*
- *Arrange to assess progress and, if necessary, make further changes in the future*

# Bone Health and Falls Assessment

- 1. Bone Health
     1. Weight (kg): Height (cm):

Waist (cm) BMI:

- - 1. History
- Known osteoporosis
- Fragility fracture
- Current use or frequent recent use of oral or systemic glucocorticoid
- History of falls
- Family history of hip fracture
- Other cause of secondary osteoporosis
- Low body mass index (BMI) (less than 18.5 kg/m2)
- Smoking
- Alcohol intake more than 14 unites per week in women and 21 unit per week in men
  - 1. FRAX Score: (<https://www.sheffield.ac.uk/FRAX/tool.aspx>)
    2. Vitamin D *(check levels with risk factors such as, bones disease, low bone density, osteoporosis, Paget’s disease, Hyperparathyroidism)*

- 1. Falls assessment
- Is there a history of any fall in the previous year? Y🞎 N❑
- Is the patient / client on four or more medications per day? Y🞎 N❑
- Does the patient / client have a diagnosis of stroke or Parkinson's disease? Y🞎 N❑
- Does the patient / client report any problems with his/ her balance? Y🞎 N❑
- Is the patient/client unable to rise from a chair of knee height without using their arms? Y🞎 N❑

*If there is appositive response to three or more of the questions, then consider further assessment or fall prevention referral. Consider which referral would be most appropriate given the patient’s needs and local resources. (see* [*http://www.1000livesplus.wales.nhs.uk/sitesplus/documents/1011/FRATtool.pdf*](http://www.1000livesplus.wales.nhs.uk/sitesplus/documents/1011/FRATtool.pdf) *)*

# Function, Social and Environmental Assessment

- 1. Nottingham Extended ADL Scale

| The following questions are about everyday activities. Please answer by ticking ONE cell for each question. Please record what you have ACTUALLY done in the last few weeks. | | | | |
| --- | --- | --- | --- | --- |
|  |  |  |  |  |
| Did You …… | Not at all = 0 | With Help = 0 | On your own with difficulty = 1 | On your own = 1 |
| 1. Walk around outside? |  |  |  |  |
| 2. Climb stairs? |  |  |  |  |
| 3. Get in and out of a car? |  |  |  |  |
| 4. Walk over uneven ground? |  |  |  |  |
| 5. Cross roads? |  |  |  |  |
| 6. Travel on public transport? |  |  |  |  |
| 7. Manage to feed yourself? |  |  |  |  |
| 8. Manage to make yourself a hot drink? |  |  |  |  |
| 9. Take hot drinks from one room to another? |  |  |  |  |
| 10. Do the washing up? |  |  |  |  |
| 11. Make yourself a hot snack? |  |  |  |  |
| 12. Manage your own money when out? |  |  |  |  |
| 13. Wash small items of clothing? |  |  |  |  |
| 14. Do your own housework? |  |  |  |  |
| 15. Do your own shopping? |  |  |  |  |
| 16. Do a full clothes wash? |  |  |  |  |
| 17. Read newspapers or books? |  |  |  |  |
| 18. Use the telephone? |  |  |  |  |
| 19. Write letters? |  |  |  |  |
| 20. Go out socially? |  |  |  |  |
| 21. Manage your own garden? |  |  |  |  |
| 22. Drive a car? |  |  |  |  |
| Scoring:  0 – with help; no  1 – on my own; on my own with difficulty |  |  |  |  |
| Total Score: |  | | | |
| Comments: | | | | |

**Consider referring to physiotherapist if walking aids are in need of review**.

- 1. Home Hazard Checklist

| Tick appropriate box |  |  |
| --- | --- | --- |
| **Question** | Yes | No |
| **Outdoor:**  a. Is your front path clear of objects and in good repair? |  |  |
| b. Are the paths, steps and entrances to your house well lit? |  |  |
| c. Do you find it difficult to unlock or lock your outside doors? |  |  |
| d. Do you have rails to hold onto at your front and back doors? |  |  |
| e. Do you empty the household rubbish into the outside bin? |  |  |
| Comments: | | |
| **Stairs:**  a. Do you switch the light on when you use the stairs at night? |  |  |
| b. Can you clearly see the edges of the steps? |  |  |
| c. Are the coverings on the steps in good condition? |  |  |
| d. Is anything stored in the stairway, even temporarily? |  |  |
| e. Do you have a handrail/bannister? |  |  |
| Comments: | | |
| **Bedroom:**  a. Do you have a light switch within reach from the bed? |  |  |
| b. Is there a telephone close to your bed? |  |  |
| c. If you have a pendant alarm, do you wear it when going to the toilet at night? |  |  |
| d. Do you have a loose rug by your bedside? |  |  |
| Comments: | | |
| **Bathroom:**  a. Do you use a non-slip mat on the bottom of the bath or shower tray |  |  |
| b. Do you sit on a bath board or shower chair? |  |  |
| c. Is there a grab rail to help you to get into and out of the bath/shower? |  |  |
| Comments: | | |
| **Toilets:**  a. Do you have any equipment to help you get on/off the toilet, |  |  |
| b. When you use the toilet, do you hold onto the sink, bath or towel rail? |  |  |
| c. Do you walk into the toilet with your walking aid? |  |  |
| Comments: | | |
| **Kitchen:**  a. Do you store items that you regularly use in high cupboards? |  |  |
| b. Do you need to bend over to reach for the fridge/oven? |  |  |
| c. Is there a continuous working surface so you can slide a saucepan or kettle to avoid lifting it up? |  |  |
| d. What type of floor covering do you have in the kitchen? Lino  Carpet  Loose rugs |  |  |
|  |  |  |
|  |  |  |
| Comments: | | |
| **Clothing and footwear:**  a. Do you ever wear clothes with trailing hems or cords? |  |  |
| b. Do you wear long nightgown or flared pyjamas? |  |  |
| c. What type of footwear do you use at home? |  |  |
| Supportive slippers with non-slip soles |  |  |
| Slippers with no back |  |  |
| Outdoor shoes |  |  |
| Barefoot |  |  |
| Comments: | | |
| **Miscellaneous**:  a. Do you have any loose rugs on top of polished floors, lino and carpet? |  |  |
| b. Are there any wires trailing across the floor. |  |  |
| c. Do you have a letterbox cage to avoid stooping to pick up the mail? |  |  |
| Comments: | | |
| **Additional information and outcome:** | | |

- - 1. Place of residence

- - 1. Marital Status
    2. Benefits received
    3. Informal Support/carer
- Living with carer
- Carer lives nearby
- Carer lives at a distance
- Does not have a carer
- Lives alone
- Informal carer
- Has an older carer
- Has a paid carer
- Has voluntary carer
  - 1. Patient consent to given to contact carer about care Y🞎 N❑
    2. Carer assessment
- Referral for assessment of needs of carer
- Carer assessment declined
  - 1. Is patient a carer themselves Y🞎 N❑
    2. Formal Support

Support services in place

- Under care of social services
- Received help from voluntary agency
- Attending day care

Support services required

- Home help needed
- Needs an advocate
- Referral to social services
- Referral to telecare
- Referral to voluntary service
  - 1. Care provision regimes
    2. Safeguarding concern
    3. Venerable adults
  1. Driving

Driving status

- Patient advised about driving
- Education: Implication to license
- Patient advised to inform DVLA
- Patient advised to inform insurance company

# Mobility and Balance

- 1. Timed Up and GO
  2. Balance
- Problem with balance
- Balance normal

# Psychological Health

- 1. Memory
     1. Initial memory assessment:

Ask the patient “Have you been more forgetful in the last 12 months to the extent that it has affected their daily life?’ *(For example, difficulty using the phone, managing shopping lists, using money, managing their medication, driving, etc)*

- - - Initial memory assessment declined Y🞎 N❑
    - Memory recall normal Y🞎 N❑

***If no reported memory problems on initial questioning please record Memory Recall Normal, otherwise continue to GPCog***

- - 1. GP-cog test:

*Unless specified, each question should only be asked once*

Step 1: Patient assessment

| **Name and Address for subsequent recall test** | | |
| --- | --- | --- |
| 1. “I am going to give you a name and address. After I have said it, I want you to repeat it. Remember this name and address because I am going to ask you to tell it to me again in a few minutes: John Brown, 42 West Street, Kensington”. (Allow a maximum of 4 attempts). | | |
| (Please tick appropriate box) | Correct | Incorrect |
| **Time orientation** | | |
| 2. What is the date? (exact only) |  |  |
| **Clock Drawings – Use blank page** | | |
| 3. Please mark in all the numbers to indicate the hours of a clock (correct spacing required) 4. Please mark in hands to show 10 minutes past eleven o’clock (11:10) |  |  |
| 3. Please mark in all the numbers to indicate the hours of a clock (correct spacing required) 4. Please mark in hands to show 10 minutes past eleven o’clock (11:10) |  |  |
| **Information** | | |
| 5. Can you tell me something that happened in the news recently? (Recently = in the last week. If a general answer is given, e.g. “war”, “lots of rain”, ask for details. Only specific answers scores). |  |  |
| **Recall** | | |
| 6. What was the name and address I asked you to remember? | | |
| John |  |  |
| Brown |  |  |
| 42 |  |  |
| West Street |  |  |
| Kensington |  |  |
| (To get a total score, add the numbers of items answered correctly Total Correct (Score out of 9) | **/9** | |
| **If patient scores 9, no significant cognitive impairment and further testing not necessary.** | | |
| **If patient scores 5 – 8, more information required. Proceed with Step 2, carer section** | | |
| **If patient scores 0 - 4, cognitive impairment is indicated. Refer to Memory Clinic** | | |

Step 2: Carer Interview

| Carers relationship to patient i.e. carer is the  patient’s: |  | | | |
| --- | --- | --- | --- | --- |
| These 6 questions ask how the patient is compared to when s/he was well, say 5 -10 years ago Compared to a few years ago: | | | | |
| (Please tick appropriate box) | Yes | No | Don’t know | N/A |
| Does the patient have more trouble remembering things that have happened recently than s/he used to? |  |  |  |  |
| Does he or she have more trouble recalling conversations a few days later? |  |  |  |  |
| When speaking, does the patient have more difficulty in finding the right words or tend to use the wrong words more often? |  |  |  |  |
| Is the patient less able to manage money and financial affairs? (e.g. paying bills, budgeting) |  |  |  |  |
| Is the patient less able to manage his or her medication independently? |  |  |  |  |
| Does the patient need more assistance with transport? (either private or public) If the patient has difficulties due only to physical problems e.g. bad leg, tick ‘no’ |  |  |  |  |
| Scores | |  |  |  |
| To get a total score, only add the number of items answered ‘no’, don’t know or Not Applicable | | | | |
| **Total Score (out of 6)** | | **/6** | | |
| If patient scores 0 – 3, cognitive impairment is indicated. Refer to Memory Clinic | | | | |

- - 1. Any history of Delirium Y🞎 N❑

1. Mood
   - 1. Initial mood assessment

Ask the patient

- - - 1. During the last month, have you often been bothered by feeling down, depressed or hopeless?
      2. During the last month, have you often been bothered by having little interest or pleasure in doing things?
      3. Do you have problem with feeling anxious?
      4. Do you worry about the future and what it might hold?

*If the patient answers YES to any of the above questions proceed with the GDS-15 below*

|  | (Please **circle** appropriate answer) | Yes | No |
| --- | --- | --- | --- |
| 1 | Are you basically satisfied with your life? | yes | **NO** |
| 2 | Have you dropped many of your activities and interests? | **YES** | no |
| 3 | Do you feel that your life is empty? | yes | **NO** |
| 4 | Do you often get bored? | **YES** | no |
| 5 | Are you in good spirits most of the time? | yes | **NO** |
| 6 | Are you afraid that something bad is going to happen to you? | **YES** | no |
| 7 | Do you feel happy most of the time? | yes | **NO** |
| 8 | Do you often feel helpless? | **YES** | no |
| 9 | Do you prefer to stay at home, rather than go out and do new things? | **YES** | no |
| 10 | Do you feel you have more problems with memory than most? | **YES** | no |
| 11 | Do you think it is wonderful to be alive? | yes | **NO** |
| 12 | Do you feel pretty worthless the way you are now? | **YES** | no |
| 13 | Do you feel full of energy? | yes | **NO** |
| 14 | Do you feel that your situation is hopeless? | **YES** | no |
| 15 | Do you think that most people are better off than you are? | **YES** | no |
|  | **15-item GDS score** | **/15** | |
|  | *(Score 1 for answers in block capitals: 0-4 normal, 5-9 Mild depression, 10-15 More severe depression)* | | |

# Care and Support Plan

- 1. What is matter to me
     1. **What is important to me** *(this can include person’s hopes and fears, practical matters [e.g. likes the TV on, likes to be outside], family concerns, spiritual care, anything else person can think of)*
     2. **What makes life meaningful to me** (*this include values, people, pets, ways you would like those caring for you to look after your spiritual and emotional needs, and anything else you want)*
     3. **What is working and not working for me** *(This helps us to understand what is working that we need to ensure keeps working, and any treatment/plans don’t inadvertently change what is going well. What is the beginning of setting priorities? It is also a way to later check that interventions and treatment are changing what is not working, so contributes to quickly assurance/evaluation.)*
     4. **What I want in the future** *(it is important to know what the person is hoping to d in the future. They define the future in relation to how far they feel comfortable looking ahead [for some persons just a month])*
     5. **What is bothering me the most, my priorities for change** *(From what is not working we want to understand the person’s top priorities for change)*
     6. **What I have tried already, and my ideas** *( this is an asset based approach and therefore we assume that people have already been trying to address what is not working or have ideas about how to do this)*
     7. **How I would like information** *(this is to help decision making and information sharing, Rather than one-size fits all communication we want to learn what works best for the person, for example emails, phone calls, letters, visits. )*
     8. **What I would like professional to know about me and my health** *(this could be the person’s health history from their perspective, or other key information that they want to share.)*
  2. Problem list
  3. Referrals

Referrals made to:

- Continence nurse
- Dental Service
- Dietitian
- Elderly falls prevention clinic
- Falls assessment
- Community falls service
- Community Matron
- Hearing therapy service
- Community mental health team
- Mental health intermediate care
- Pharmacist
- Geriatrician
- Occupational therapist
- Social worker
- EDAS
- Other Specify below
  1. Advanced care plan ( add extra rows if needed)

| **Goal (I would /would not want: )** | **Actions** |
| --- | --- |
|  |  |
|  |  |
|  |  |
|  |  |
