# Supplementary material for: Comprehensive geriatric assessment delivered by advanced nursing practitioners within primary care setting: a mixed-methods pilot feasibility randomised controlled trial
Source: BMC Geriatr. 2023 Aug 24;23:513. doi: 10.1186/s12877-023-04218-0 (PMC10463370; doi:10.1186/s12877-023-04218-0)
Supplement: Supplementary file 3 — Additional file 3. [file 12877_2023_4218_MOESM3_ESM.docx]

# **Comprehensive Geriatric Assessment Delivered by Advanced Nursing Practitioners within Primary Care Setting: A Mixed-methods Pilot Feasibility Randomised Controlled Trial**

# **Additional File 3: Interview schedule**

The purpose of the interviews is to explore the older adult’s perception and experience of being part of the research project. Participants will be asked about their perceived challenges, strength and benefits of the assessment, care and support plan. Interviewees will be asked questions on the following themes:

**Themes**

Expectations and perception

- What were your expectations from the research before the study?
- How do you feel about the programme?
- Did you have any previous experience of the frailty assessment and or CGA programme?
- Has the programme responded to your (ongoing) health needs and concerns?
- How do you perceive the interaction between you and the nurse, ACP and specialists?
- Did you get all treatments that you perceived you would get through the programme? Followed by probing questions about medical physical, Mental, Function, Environment and Social domains.

Quality and benefits

- How would you rate the quality of care received on a scale of 0 to 10, with 0 being the worst quality and 10 being the best quality? Why?
- How would you compare your health status before and after the programme? Followed by probing questions about medical physical, Mental, Function, Environment and Social domains.
- How do you rate your independence in activities of daily living before and after the research?

Barriers, challenges and problems

- Were there any challenges or problems with assessment and care planning which stopped you from full benefit from the research?
- Were you referred for further assessment by other specialities? If so, were there any barriers or problems you faced?

Recommendation for an effective and more feasible CGA programme

- What do you think is needed to overcome barriers and problems with the programme?
- If what you experienced during this research becomes normal practice what would you think could improve it?
- What would be the three main messages that you would like to give to the practitioners, nurses and your GP Practice?
