# Supplementary material for: Comprehensive geriatric assessment delivered by advanced nursing practitioners within primary care setting: a mixed-methods pilot feasibility randomised controlled trial
Source: BMC Geriatr. 2023 Aug 24;23:513. doi: 10.1186/s12877-023-04218-0 (PMC10463370; doi:10.1186/s12877-023-04218-0)
Supplement: Supplementary file 4 — Additional file 4. [file 12877_2023_4218_MOESM4_ESM.docx]

# Comprehensive Geriatric Assessment Delivered by Advanced Nursing Practitioners within Primary Care Setting: A Mixed-methods Pilot Feasibility Randomised Controlled Trial

# Additional File 4: CGA&CSP baseline clinical characteristics

# Contents

[1) Summary 2](#_Toc116478479)

[2) Physical health 2](#_Toc116478480)

[3) Medication 3](#_Toc116478481)

[4) Bone health and falls 3](#_Toc116478482)

[5) Function, social, environment 3](#_Toc116478483)

[6) Mobility and balance 4](#_Toc116478484)

[7) Care and support plan 4](#_Toc116478485)

[8) Referrals at baseline, interim and follow-up. 4](#_Toc116478486)

# Summary

The summary of problems identified included vision problems (n=9), Polypharmacy (i.e ≥ 5 medications; n= 30), fall history (n=13), difficulty with balance (n=17), risk of fracture due to osteoporosis (n=24). Besides, major problems identified were diabetes, fractures, diverticulitis, cancer, osteoarthritis/arthritis, hypertension and high blood pressure. Other problems included allergies/sensitivities such as hay fever, rashes and intestine/indigestion problems.

As part of the care and support planning participants were asked to answer questions such as ‘What is important to me’ and ‘what makes life meaningful for me’. The majority of participants stated that family and friends as being the most important that makes life meaningful. The importance of independent living and leisure activities were also emphasised, and therefore, to be more active, in good health and being at home were what the participants wished for the future.

Referrals were made for 18 (53%) participants at the initial CGA session to GP (n=5), ANP (n=8), Occupational Therapy (n=1), community Matron (n=2), Falls prevention (n=2) and care co-ordinator (n=1). Also, referrals were made for 9 (24%) and 13 (35%) participants after the interim and final follow up CGA sessions, respectively.

# Physical health

Table 1 presents the baseline clinical characteristics for physical health. This includes the MRC Breathlessness scale score which had an interquartile range of *md=*3. A central nervous system observation in where all participants performed well (fully conscious n=14, nothing abnormal detected n=20). Oral health was discussed and only n=2 participants needed advice. The participants' alcohol intake was discussed and n=15 reported to be teetotal, n=11 light drinker, n=1 a moderate drinker, n=6 reported to drink 1-2 units per week and n=1 reported to drink 1-2 units per month. Weight management was discussed, however only n=5 participants needed advice. Sleep patterns were reported as n=21 participants having more than eight hours of sleep per nights, n=5 reported to have six to eight hours per night and n=8 reported to get less than six. Smoking was also examined and n=17 reported to have never smoked tobacco and n=14 reported being ex-smokers. There was n=2 participants who reported smoking and n=1 participant was given a brief smoking intervention. The participants' current vision health was discussed and there was n=27 participants who wore glasses (n=5 long sighted, n=15 short sighted, n=4 bifocals, n=3 varifocals). There was n=9 participants who needed a referral to an opticians. The final physical health category discussed with the participant was hearing problems. There was n=19 participants who report no hearing problem at all and n=13 participants who wore hearing aids. The remaining n=3 declined to wear hearing aids.

# Medication

Table 2 presents the baseline clinical characteristics for medication. Participants last medication review was reported and who completed this. There was n=10 review by a GP, n=5 Pharmacist, n=7 Practice Nurse, n=3 Consultant. There was n=8 who did not need medication reviews. There was n=29 participants who self-administered their medication and n=5 participants where it was managed for them. Prescriptions were collected by pharmacist (n=12), family member (n=2), patient (n=5). For n=15 participants it is unknown who collects medication. The number of medication per participant was calculated and n=4 participants had less than or equal to four, n=26 participants had five to ten and n=4 had more than or equal to 11.

# Bone health and falls

Table 3 presents the baseline clinical characteristics for bone health and falls. Participants were screened for their history of falls. There was n=9 who had no history of falls, n=13 who did but not in the last year and n=2 who had had a fall in the last year. Participants were than assessed for their ability to rise from chair of knee height without using their arms. There was n=10 who were able to do this and n=21 who were unable to do this. Participants FRAX score indicated n=4 was low risk, n=8 was osteoporotic, n=16 was major osteoporotic, and n=2 was out of range.

# Function, social, environment

Table 4 presents the baseline clinical characteristics for function, social, environment. The Nott ADL score had an interquartile range of *md=*8. The participants marital status was recorded as married/partner (n=17), widow/widower (n=14), divorced (n=2) or single (n=1). The participant was asked about informal carer support. There were n=16 participants who did not have a carer, n=3 had a privately paid carer and n=3 received care provided by social services. There was n=3 participants who had support from a wider family member and n=9 was living with their informal carer.

# Mobility and balance

Table 5 presents the baseline clinical characteristics for mobility and balance. This assessment reported that n=16 balance was normal, n=17 had difficulties with balance.

# Care and support plan

Table 6 presents the baseline clinical characteristics for acre and support plan. In this section the participants were asked what was important to them. The participants reported that partner/family/friends (n=20) were important, independence (n=6), getting help (n=1), leisure activities (n=5). The participants were also asked what makes life meaningful. They reported, family (n=24), faith (n=2), leisure activities (n=4), friends (n=8), pet (n=2), living independently (n=2). Finally participants were asked what they wished for the future. This included, to be more active (n3), stay active (n=4), to manage health (n=2), to have good health (n=8), to be able to stay at home (n=4), to have regular contact with the GP (n=1), to have home visits (n=1), to have the acre plan reviewed (n=1), watch their grandchildren grow (n=1). There was also n=1 participant who said they were happy at present, n=1 who stated they have planned for their funeral and n=1 stated that they want their scheduled operation.

# Referrals at baseline, interim and follow-up.

Table 7 presents the referrals made as part of the CGA intervention at the baseline (n=18), interim (n=9) and follow-up (n=13) stages of its delivery. The referrals were made to the GP (n=5 baseline, n=1 interim, n=8 follow-up), Advance Nurse Practitioner (n=8 baseline, n=3 interim, n=2 follow-up), Occupational therapists (n=1 baseline, n=1 interim), Community Matron (n=2 baseline), falls prevention (n=2 baseline, n=1 interim), care co-ordinator (n=1 baseline, n=5 follow-up).

Table 1:Baseline clinical characteristics of CGA (physical Health)

| **CGA Intervention (1. Physical Health)** | |
| --- | --- |
|  | **Baseline** |
| **Parameter** | **n (%) *IQR** |
| MRC Breathlessness Scale Score | *3 |
| Central Nervous System Observation |  |
| - GCS 15 (fully conscious) | 14 (38) |
| - Nothing abnormal detected | 20 (54) |
| Oral health advice needed |  |
| - No | 27 (73) |
| - Yes | 2 (5) |
| - n/a | 5 (14) |
| Alcohol status |  |
| - Teetotaller | 15 (41) |
| - Light drinker 1-2 unit per day | 11 (30) |
| - Moderate drinker 3-6 u/day | 1 (3) |
| - 1-2 units a week | 6 (16) |
| - 1-2 units per month | 1 (3) |
| Weight management advice needed |  |
| - No | 29 (78) |
| - Yes | 5 (14) |
| Sleep problem |  |
| - 6< hours | 8 (22) |
| - 6-8 hours a night | 5 (14) |
| - 8+ hours | 21 (57) |
| Smoking |  |
| - Ex-smoker | 14 (38) |
| - Never smoked tobacco | 17 (46) |
| - Smoker | 2 (5) |
| brief intervention given | 1 |
| - n/a | 1 (3) |
| Vision |  |
| - None | 6 (16) |
| - Glasses | 27 (79) |
| Long sighted | 5 (14) |
| Short sighted | 15 (41) |
| Bifocal | 4 (11) |
| Varifocals | 3 (8) |
| - Cataract | 1 (3) |
| - Glaucoma | 2 (5) |
| - Registered blind | 1 (3) |
| Optician referral needed |  |
| - Yes | 9 (24) |
| Hearing Problems |  |
| - None | 19 (49) |
| - Wears a hearing aid | 13 (35) |
| - Declines to wear hearing aid | 3 (8) |
| Total | 34 |

Table 2: Baseline clinical characteristics of CGA (Medication)

| **CGA Intervention (2. Medication)** | |
| --- | --- |
|  | **Baseline** |
| **Parameter** | **n (%)** |
| Who last reviewed patients medication |  |
| - GP | 10 (27) |
| - Pharmacist | 5 (14) |
| - Practice Nurse | 7 (19) |
| - Consultant | 3 (8) |
| - Could not remember | 1 (3) |
| - N/A | 8 (22) |
| Administration of medication |  |
| - Does not manage medication | 5 (14) |
| - Self-administration of medication | 29 (78) |
| Prescriptions collected by |  |
| - Pharmacist | 12 (32) |
| - Family member | 2 (5) |
| - Patient | 5 (14) |
| - Unknown | 15 (41) |
| Number of medications per patient |  |
| - ≤4 | 4 (11) |
| - 5 to 10 | 26 (70) |
| - ≥11 | 4 (11) |
| Total | 34 |

Table 3: Baseline clinical characteristics of CGA (Bone health and falls)

| **CGA Intervention (3. Bone health and Falls)** | |
| --- | --- |
|  | **Baseline** |
| **Parameter** | **n (%)** |
| History of falls |  |
| - None | 9 (24) |
| - Yes (not in last year) | 13 (35) |
| - Yes (within the last year) | 2 (5) |
| - Unknown | 10 (27) |
| Unable to rise from chair of knee height without using arms |  |
| - Yes | 10 (27) |
| - No | 21 (57) |
| - N/A | 3 (8) |
| FRAX Score |  |
| - Low Risk | 4 (12) |
| - Osteoporotic | 8 (24) |
| - Major Osteoporotic | 16 (47) |
| - Out of range | 2 (6) |
| - N/A | 4 (12) |
| Total | 34 |

Table 4: Baseline clinical characteristics of CGA (Function, social, environment)

| **CGA Intervention (4. Function, social, environment)** | |
| --- | --- |
|  | **Baseline** |
| **Parameter** | **n (%) *IQR** |
| Nott ADL Score |  |
| - STDEV | *8 |
| Marital status |  |
| - Married/partner | 17 (46) |
| - Widow/Widower | 14 (38) |
| - Divorced | 2 (5) |
| - Single | 1 (3) |
| Informal support-carer |  |
| - Does not have a carer | 16 (43) |
| - Has a privately paid carer | 3 (8) |
| - Has help from wider family | 3 (8) |
| - Living with informal carer | 9 (24) |
| - Under the care of social services | 3 (8) |
| Total | 34 |

Table 5: Baseline clinical characteristics of CGA (Mobility and balance)

| **CGA Intervention (5. Mobility and balance)** | |
| --- | --- |
|  | **Baseline** |
| **Parameter** | **n (%)** |
| Balance |  |
| - Normal | 16 (43) |
| - Difficulties with balance | 17 (46) |
| - N/A | 1 (3) |
| Total | 34 |

Table 6: Baseline clinical characteristics of CGA (Care and support plan)

| **CGA Intervention (6 Care and support plan)** | |
| --- | --- |
|  | **Baseline** |
| **Parameter** | **n (%)** |
| What is important to me |  |
| - Partner/Family/Friends | 20 (54) |
| - Independence | 6 (16) |
| - Getting help | 1 (3) |
| - Leisure activities | 5 (14) |
| - Did not want to answer | 2 (5) |
| What makes life meaningful |  |
| - Family | 24 (65) |
| - Faith | 2 (5) |
| - Leisure activities | 4 (11) |
| - Friends | 8 (22) |
| - Pet | 2 (5) |
| - Living independently | 2 (5) |
| - Do not know/did not answer | 5 (8) |
| Wish for the future |  |
| - To be more active | 3 (8) |
| - Stay active | 4 (11) |
| - To manage health | 2 (5) |
| - Good health | 8 (22) |
| - Stay at home | 4 (11) |
| - Regular contact with GP | 1 (3) |
| - Home visits | 1 (3) |
| - Review care plan | 1 (3) |
| - Watch Grandchildren grow up | 1 (3) |
| - Happy at present | 2 (5) |
| - Has planned for funeral | 1(3) |
| - To have an operation | 2 (5) |
| Total | 34 |

Table 7: Comparison of CGA Referrals during baseline, interim and final assessments

| **CGA Intervention (7.Referrals)** | |  |  |
| --- | --- | --- | --- |
|  | **Baseline** | **Interim** | **Final Follow up** |
| **Parameter** | **n (%)** |  |  |
| Referrals |  |  |  |
| - Yes | 18 (53) | 9 (24) | 13 (35) |
| - No | 16 (47) | 24 (65) | 19 (51) |
| Type of referral |  |  |  |
| - GP | 5 | 1 | 8 |
| - ANP | 8 | 3 | 2 |
| - OT | 1 | 1 | - |
| - Community Matron | 2 | - | - |
| - Falls prevention | 2 | 1 | - |
| - Care co-ordinator | 1 | - | 5 |
| Total patients | 34 | 33 | 32 |
